# Integrated approach in the control and management of skin neglected tropical diseases in Lalo, Benin

**DOI:** 10.1371/journal.pntd.0006584

**Published:** 2018-06-25

**Authors:** Yves Thierry Barogui, Gabriel Diez, Esai Anagonou, Roch Christian Johnson, Inès Cica Gomido, Hermione Amoukpo, Zoulkifl Salou Bachirou, Jean Gabin Houezo, Raoul Saizonou, Ghislain Emmanuel Sopoh, Kingsley Asiedu

**Affiliations:** 1 Centre de dépistage et de Traitement de l’Ulcère de Buruli de Lalo, Ministry of Health, Lalo, Benin; 2 Projects Department, Fondation Anesvad, Bilbao, Espagne; 3 Programme National de Lutte contre la Lèpre et l’Ulcère de Buruli, Ministry of Health, Cotonou, Benin; 4 Centre Inter Facultaire de Formation et de Recherche en Environnement pour le Développement Durable, Université d’Abomey Calavi, Abomey Calavi, Benin; 5 Centre de dépistage et de Traitement de l’Ulcère de Buruli d’Allada, Ministry of Health, Allada, Benin; 6 Department of Neglected Tropical Diseases, World Health Organization, Cotonou, Benin; 7 Institut Régional de Santé Publique, Université d’Abomey Calavi, Ouidah, Benin; 8 Department of Neglected Tropical Diseases, World Health Organization, Geneva, Switzerland; University of Tennessee, UNITED STATES

## Abstract

**Background:**

Neglected Tropical Diseases (NTDs) are a group of several communicable diseases prevalent in the tropical and subtropical areas. The co-endemicity of these diseases, the similarity of the clinical signs, and need to maximize limited financial and human resources have necessitated implementation of integrated approach. Our study aims to share the lessons of this integrated approach in the fight against Buruli ulcer (BU), leprosy and yaws in a rural district in Benin.

**Methods:**

It is a cross-sectional study using a single set of activities data conducted from May 2016 to December 2016. Health workers and community health volunteers involved in this study were trained on integrated approach of the Buruli ulcer, leprosy and yaws. Village chiefs were briefed about the activity. The trained team visited the villages and schools in the district of Lalo in Benin. After the education and awareness raising sessions, all persons with a skin lesion who presented voluntarily to the team were carefully examined in a well-lit area which respected their privacy. Suspected cases were tested as needed. The socio-demographic information and the characteristics of the lesions were collected using a form. A descriptive analysis of the epidemiological, clinical and laboratory variables of the cases was made using Excel 2013 and SPSS version 22.00.

**Principal findings:**

In the study period, 1106 people were examined. The median (IQR) age of those examined was 11 (8; 27) years. Of 34 (3.1%) suspected BU cases, 15 (1.4%) were confirmed by PCR. Only three cases of leprosy were confirmed. The 185 (16.7%) suspected cases of yaws were all negative with the rapid test. The majority of cases were other skin conditions, including fungal infections, eczema and traumatic lesions.

**Conclusion:**

The integrated approach of skin NTD allows optimal use of resources and surveillance of these diseases. Sustaining this skin NTD integrated control will require the training of peripheral health workers not only on skin NTD but also on basic dermatology.

## Author summary

Neglected Tropical Diseases are a group of several communicable diseases prevalent in the tropical and subtropical areas. The co-endemicity of these diseases, the similarity of the clinical signs, need to maximize limited financial and human resources have necessitated the integrated approach. Our study aims to share the lessons learned from the integrated approach in the fight against Buruli ulcer, leprosy and yaws in a rural district in Benin. In this study, health workers, community health volunteers involved in this study were trained on integrated approach of the Buruli ulcer, leprosy and yaws. The trained team visited the villages and schools in the district of Lalo in Benin. After the education and awareness raising session, all persons with a skin disease who presented voluntarily to the team were carefully examined in a well-lit area which respected their privacy. Of 1106 people with skin diseases, 15 (1.4%) were Buruli ulcer and three (0.3%) were leprosy. The majority of cases were other skin conditions. Sustaining this skin Neglected Tropical Diseases integrated control will require the training of peripheral health workers not only on skin Neglected Tropical Diseases but also on basic dermatology to deal with other common skin diseases.

## Introduction

Neglected Tropical Diseases (NTD) are a diverse group of communicable diseases prevalent in tropical and subtropical areas. They are endemic in 149 countries. At least 100 of these countries have two or more endemic NTD. At least 30 of these countries have 6 or more endemic NTD [1,2]. They affect more than a billion people and cost developing economies billions of dollars every year. The socio-economic consequences are enormous [1]. The populations most affected by NTD are the poor [2,3].

Of the 20 NTD, most of them are endemic in Africa. Case management interventions are implemented for specific NTD by the World Health Organization Regional Office for Africa. Eight of those NTD have cutaneous manifestations: Buruli ulcer (BU), leprosy, yaws, cutaneous leishmaniasis, lymphatic filariasis morbidity, scabies, ectoparasites and mycetoma. WHO has recently published an integrated training manual on skin NTDs to help countries move forward in this direction [4]. Worldwide, progress has been made in the fight against these diseases. So by 2020, the objectives are respectively the eradication of yaws, the elimination of leprosy and the control of BU [5–7].

The co-endemicity of certain diseases; the similarity of the clinical signs as well as the scarcity of financial, human and temporal resources called for an integrated management of these diseases [8,9]. So at the May 2013 World Health Assembly and at the September 2013 WHO Regional Committee for Africa, two resolutions were adopted (WHA 66.12 AFR / RC63. R6), both of which recommending the integration of NTD management programs [3,10].

BU is endemic in southern Benin; whereas leprosy is endemic in both southern and northern Benin. Yaws outbreaks were reported in Benin and were effectively treated in the 1980s [11,12] but surveillance was not maintained. Onchocerciasis, lymphatic filariasis, loa loa, schistosomiasis, soil-transmitted helminthiasis, African Human Trypanosomiasis, dracunculiasis, trachoma, leprosy and Buruli ulcer are relevant NTDs in Benin [13].

Taking into account the 2013 resolution of the World Health Assembly, we set out to develop the integrated screening of BU, leprosy and yaws in the district of Lalo in southern Benin. The goal of this study is to share the lessons learned during this experience of the integrated management of three skin NTD in a rural district of Benin.

## Methods

### Ethical consideration

This study using a single set of activities data, was approved and authorized by the institutional review board of the National Program against Leprosy and Buruli Ulcer of Benin (nr013/PNLLUB/SA). It was also approved and authorized by the regional health authorities of Lalo (nr20/16/BZ-KTL/SA) as well as by the chiefs of the villages.

Informed consent was obtained orally from all adult participants and from parents, caretakers, or legal representatives of participants aged ≤18 years after obtaining their assessment.

For this study using a single set of activities data, verbal informed consent was necessitated given both high rates of illiteracy and the need to provide more details about the study in local languages; most participants spoke only local languages. Verbal informed consent was documented as “verbal informed consent given: yes or no” in a register.

Data were processed with strict respect for confidentiality and anonymity.

### Organization of the leprosy, Buruli ulcer and yaws control program in Benin

At the national level, the BU and leprosy management programs are already integrated since they are overseen by one national program known as the “*Programme National de Lutte contre la lèpre et l’Ulcère de Buruli”* (PNLLUB).

At the operational level, the leprosy and BU management programs are handled by different health centers.

In Benin, four specialized facilities known as “*Centre de Dépistage et de Traitement de l’Ulcère de Buruli*” (CDTUB), located in the southern part of the country ensure BU management and supervise the peripheral health centers where uncomplicated cases are managed. As for leprosy, screening and care are provided by eight specialized facilities known as “*Centre de Traitement Anti Lèpre*” (CTAL), which are spread across the country.

There is no yaws program and surveillance activities are done as part of the work of the CDTUB and CTAL, which act as sentinel sites.

### Implementation

This cross-sectional study using a single set of activities data took place from May 1^st^ 2016 to December 31^st^ 2016 in the district of Lalo. It is located in the south-west of Benin, 150 km from Cotonou, the country’s economic capital. This district has an area of 432 km^2^ [14]. It is divided into 11 sub-districts with a total population estimated at 132,964 inhabitants in 2016 [15]. BU and leprosy are endemic in this district [16]. This district has 115 primary schools, a CDTUB and each of its sub-districts has a health center. Thirty community health volunteers are actively involved in skin NTDs control in this district.

### Preparatory phase

We trained the health workers (nurses, midwives and laboratory technicians), teachers and community health volunteers in the integrated control and management of leprosy, BU and yaws. These actors had already demonstrated their skills in the control of BU [17]. The training of the health workers covered basic epidemiology, clinical diagnosis, differential diagnosis, complications, social consequences and treatment of these three diseases were discussed. The training for the community health volunteers and teachers was mainly focused on clinical diagnosis to increase their capacity to suspect cases. The training for the health workers was principally focused on clinical diagnosis, performing rapid diagnostic test for yaws and case management.

Furthermore, the heads of sub-districts and village chiefs received briefing on these diseases in order to obtain their support and involvement.

### Integrated screening

Social mobilization was carried out the day before as well as the day of the screening with the involvement of opinion leaders and village chiefs.

A schedule for the screening was made and approved by the health authorities of the area. In accordance with this schedule, the trained health workers went to villages and primary schools.

The activity started with an education and awareness raising session on BU, leprosy and yaws. Using the video projector, documentaries on BU and leprosy were shown to the participants. After that, the trained health workers and community health volunteers provided clear explanation on the disease by using the WHO posters on BU, leprosy and yaws.

After the education and awareness raising session, verbal informed consent was obtained from all persons with a skin lesion who presented themselves voluntarily to the team. Then they were carefully examined in a well-lit area which respected their privacy. The socio-demographic information and the characteristics of the lesions were collected using a form. Only the patients with skin lesions were excluded in this study.

BU lesions were classified according to the WHO categories: Category I (a single lesion with a diameter ≤ 5 cm); Category II (a single lesion with a diameter between 5 and 15 cm); Category III (a single lesion with a diameter >15 cm; multiple lesions; osteomyelitis; a lesion located in a critical area such as the eyes, breasts or genitals) [18].

Leprosy cases were classified as paucibacillary or multibacillary. Disabilities due to leprosy were classified according to WHO recommendations [19].

### Laboratory confirmation

For suspected BU cases, swabs or fine needle aspirate samples were collected and sent to the laboratory “Laboratoire de Référence des Mycobactéries” in Cotonou for confirmation by Polymerase Chain Reaction (PCR) for *IS2404*.

Suspected yaws cases were confirmed using a rapid non-treponemal and treponemal test (DPP Syphilis Screen and Confirm Assay, Chembio Diagnostic Systems, Medford, NY, USA).

Suspected cases of leprosy were referred to the nearest CTAL for confirmation.

### Patient care

Confirmed cases of BU were treated at CDTUB in Lalo in accordance with the WHO protocol [20]. Other cases of chronic ulcers were treated according to their etiology at the CDTUB in Lalo.

Leprosy cases were referred to the nearest CTAL for care.

Other dermatosis cases were treated as outpatients and the complicated cases were referred to the Departmental Hospital.

The results of this study were given to the district health authorities and the “Programme National de Lutte contre la Lèpre et l’Ulcère de Buruli” (PNLLUB) for decision-making.

### Mapping

The base maps were loaded on the DIVA-GIS program (http://www.diva-gis.org/) and completed by base maps of the *“*Programme National de Lutte contre la Lèpre et l’Ulcère de Buruli” (PNLLUB). The map was made using the software QGIS version 1.8.0.

### Statistical analysis

The data was recorded in Excel 2013 and analyzed with SPSS version 22.00. We conducted a descriptive analysis of the cases’ epidemiological, clinical and biological variables. To calculate the detection rate, the cases were compared to each sub-district’s population projection for 2016 made by the “Institut National de la Statistique et de l’Analyse Economique” of Benin [15].

## Results

**Fig 1:**
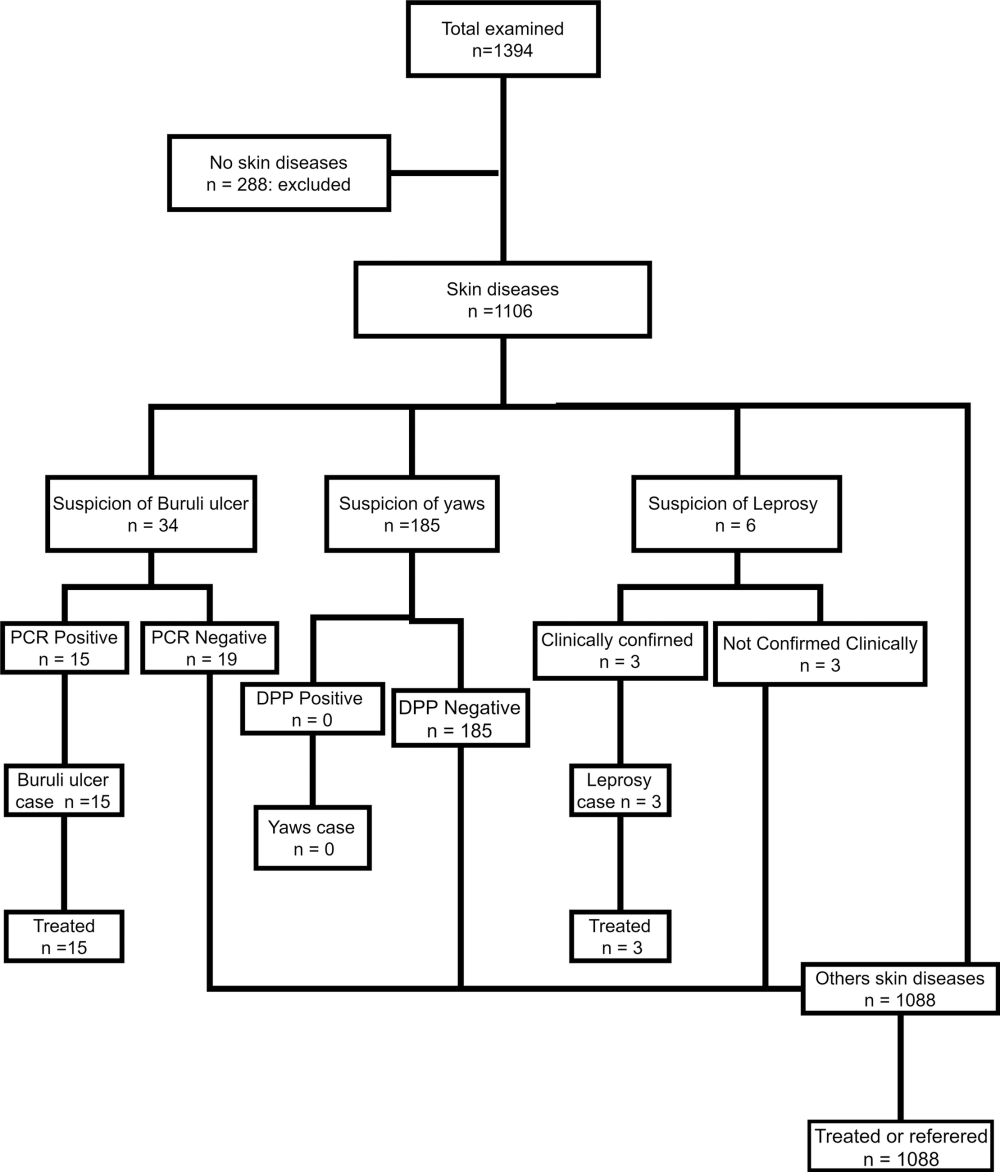
Flow-chart of cases.

A total of 1394 people were screened. There were 288 (20.7%) persons who did not have skin lesions, therefore, were excluded and 1106 (79.3%) participants who had skin lesion were included (Fig 1).

The general characteristics of the patients with skin lesions as well as the characteristics of the lesion were summarized in the Table 1. The median age (IQR) of the patients with skin lesion was 11 (8; 27) years. Just over one-third (37.0%) of those surveyed were female and 65.9% were children less than 15 years old.

**Table 1:** Description of cases (n = 1106).

Variables	Value
**Characteristics of the participants (n = 1106)**	
Median age in years (IQR)	11 (8 – 27)
Gender: Female n (%)	409 (37.0)
**Etiological classification of targeted diseases**	
Suspected Buruli ulcer n (%)	34 (3.1)
Buruli ulcer n (%)	15 (1.4)
Suspected Leprosy n (%)	6 (0.5)
Leprosy n (%)	3 (0.3)
Suspected Yaws n (%)	185 (16.7)
Confirmed Yaws n (%)	0 (0.0)
Others n (%)	881 (79.6)
**Characteristics of the patients with confirmed BU (n = 15)**	
Median age in years (IQR)	21 (10.5–32.5)
Category 3 n (%)	3 (20.0)
Limitation in movement of the joint n (%)	1 (6.7)
**Characteristics of the patients with leprosy (n = 3)**	
Age: 18–36 years	
Gender: Male (n)	3
Multibacillary leprosy (n)	3
Grade 2 disability (n)	1
**Characteristics of patients with other skin lesions (n = 1088)**	
Fungal skin infection* n (%)	533 (49.0)
Eczema n (%)	196 (18.0)
Traumatic wound / Neglected wound n (%)	96 (8.8)
Staphylococcal skin infections** n (%)	82 (7.5)
Erysipelas / Necrotizing fasciitis / Cellulitis n (%)	72 (6.6)
Vascular ulcers / Sickle cell n (%)	64 (5.9)
Tumor / Keloid n (%)	20 (1.8)
Unknown n (%)	12 (1.1)
Vitiligo n (%)	7 (0.6)
Lymphatic Filariasis n (%)	6 (0.5)

* pityriasis versicolor, tinea capitis, tinea pedis

** clinical diagnostic assessment

Of the 34 (3.1%) suspected BU cases, 15 (1.4%) were confirmed by PCR.

Only 3 cases of leprosy from the same village were confirmed clinically.

The rapid DPP test results for all 185 (16.7%) suspected yaws cases were all negative.

The majority of cases were other non-NTD skin conditions, including fungal infections (*Pityriasis versicolor*, *Tinea capitis*, *Tinea pedis*), eczema and traumatic lesions. These conditions can be reliably diagnosed clinically by trained health workers.

**Fig 2:**
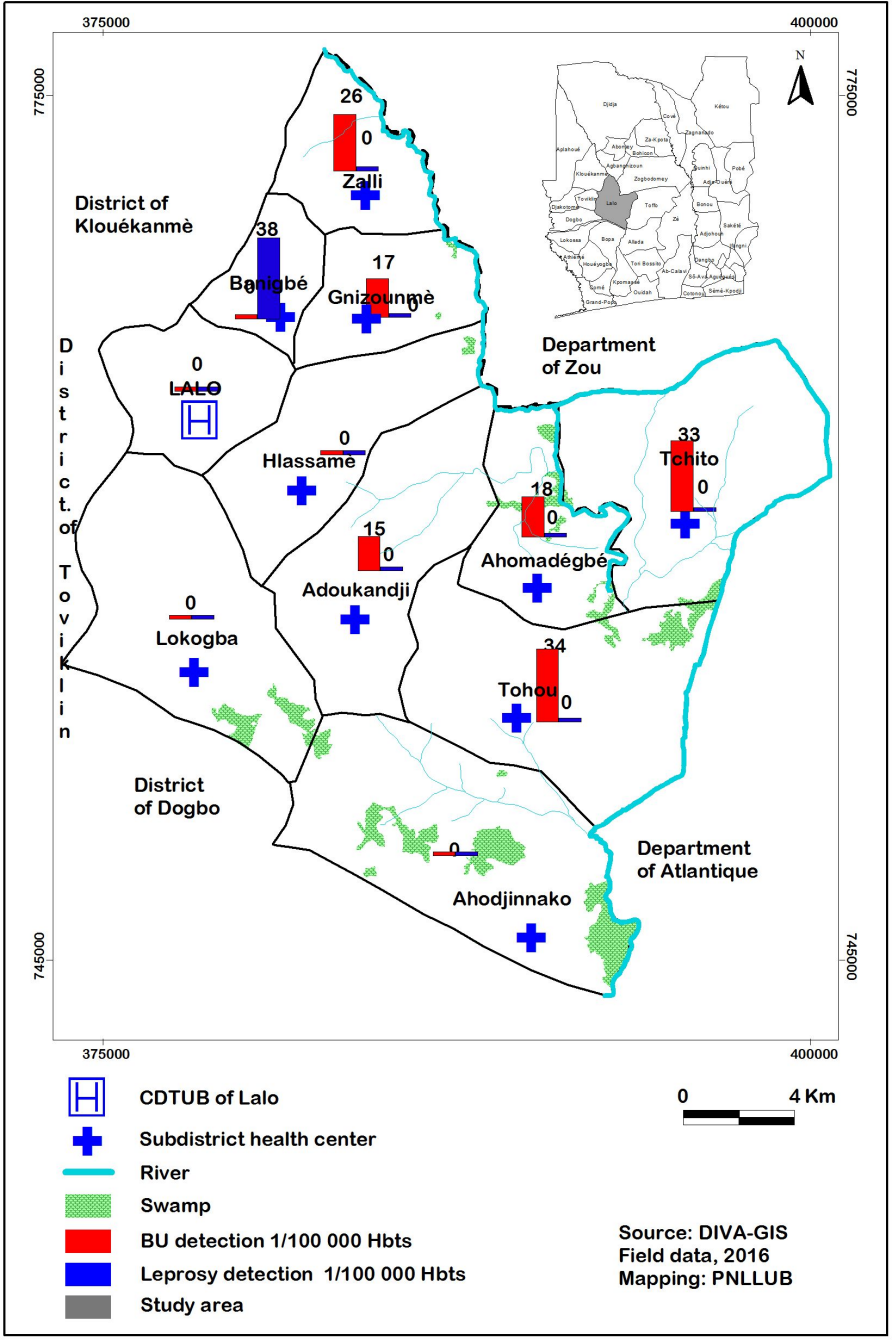
Mapping of detected Buruli ulcer and leprosy cases.

The detection rate for BU cases ranged from 0 to 34 cases per 100,000 inhabitants per sub-district. The median (IQR) age of BU patients was 21 years (10.5; 32.5). Out of the 15 confirmed BU patients, 3 (20%) had Category III lesions and only 1 patient had a limitation in movement of the joint (Fig 2).

The mapping of confirmed BU and leprosy cases is presented in Fig 2.

## Discussion

In this study, we share our experience of the integrated control and management of neglected tropical diseases in a rural district of Benin in accordance with the recommendation of resolutions WHA 66.12 AFR / RC63. R6 of the 63^***rd***^ World Health Assembly and the WHO Regional Committee for Africa [3,10]. To our knowledge, our study is one of the first attempts in West Africa to try to implement these resolutions and publish the results. In the implementation of this integrated management of NTD with cutaneous manifestations, we used the approach proposed by Mitja et al [21]. We identified co-endemic communities; we trained the team; carried out social mobilization; performed mobile medical consultations; conducted the screening and management of cases; mapping and held review meetings.

During the mobile medical consultations, all persons with skin lesions were directed to a fixed site in their village, where they were examined and treated for all skin conditions and not just for NTD with cutaneous manifestations. Mobile medical consultations are better than active door-to-door searches that require a lot of resources and are no longer recommended by the WHO for leprosy screening [22]. In addition, mobile medical consultations focused on all skin diseases have the advantage of reducing the stigma and discrimination that would result from the search of a specific NTD with cutaneous manifestations [23–26].

In our study, we screened 1,106 people to detect only 15 BU cases (1.4%) and 3 cases of leprosy (0.3%). In Malawi, Kelias et al reported 1% cases of leprosy among people with skin lesions examined in the community [27]. Our study seems to have the advantage of being efficient since, with the same resources, at least 3 types of NTDs were actively sought at the same time. Although the number of BU and leprosy cases which were confirmed and treated may seem low, these cases were detected early. Indeed, only 20% of the BU cases detected had Category III lesions and one out of the 3 leprosy cases had a Grade 2 disability. Early detection helps reduce long costly hospital stay, the functional limitations and the socio-economic consequences [28–32].

In this study, no case of yaws was confirmed. However, it is too early to state that there are no yaws in this rural district and screening should continue.

Moreover, this integrated management allowed the community-based screening team to maintain continuous monitoring; to suspect cases of yaws; to detect other NTD (lymphatic filariasis) and other dermatological conditions. This contributed to the reduction of under-reporting of NTD with cutaneous manifestations. In addition, 4 out of 5 patients (79.6%) had a dermatosis other than NTD with cutaneous manifestations. These other dermatoses were mostly fungal infections that had to be taken care of. Previous studies have shown that fungal skin infections are very common in the community [27,33]. Thus, this activity helped to solve an ethical problem as there were no exclusions: all cases were treated or referred to the dermatologist.

Furthermore, a resolution adopted by the WHO Regional Committee for Africa also recommends that African countries promote leadership in order to establish and strengthen national integrated NTD programs and foster multi-sectoral collaboration [10]. The good planning and success of the implementation of the integration result therefore from the mobilization of health system stakeholders and community actors. The ownership and adherence of the health authorities to this approach was indicated by the active participation of the PNLLUB, the health authorities, the local elected officials and the community health volunteers.

One of the limitations of this study is the absence of a dermatologist on the community screening team. In fact, for a population of more than 11 million inhabitants, Benin has less than fifteen dermatologists (less than three dermatologists for about two million people). All of them are located in the big cities. As a result, it is impossible to have a dermatologist in rural areas where NTD with cutaneous manifestations are more endemic [34–36]. Another limitation of this study is the organization of community consultations. As a matter of fact, patients who have unsightly lesions may be ashamed to appear in public [23,25,37,38]. But since the screenings were preceded by education and awareness raising sessions, the relatives of these patients at least had the information and could then discreetly take them to the referral health center for their care. In this study, we did not evaluate the cost of these activities. Future studies might focus on financial aspects.

At the end of this study, we can conclude that the integrated control and management of skin NTD allows optimal surveillance of these diseases. Sustaining this integrated management will require the training of peripheral health workers not only on skin NTD with but also on basic dermatology. We believe that our results will help other districts in Benin and other countries in the implementation of the integrated management of skin NTD in the world in general and in Africa in particular.

## Supporting information

S1 Checklist STROBE checklist. (DOC)

S1 Dataset Study dataset. (XLS)
